# Characteristics of myeloid sarcoma in mice and patients with TET2 deficiency

**DOI:** 10.3892/ol.2020.11907

**Published:** 2020-07-24

**Authors:** Jinhuan Wang, Zhaoyi Miao, Yanan Jiang, Ping Zou, Weiming Li, Xiaoqiong Tang, Yangyang Lv, Donghui Xing, Shi Chen, Fengchun Yang, Mingjiang Xu, Zeng Cao, Haitao Wang, Zhigang Zhao

Oncol Lett 19: 3789-3798, 2020; DOI: 10.3892/ol.2020.11479

Subsequently to the publication of the above article, the authors have realized that three of the four fluorescence-activated cell sorting (FACS) images in Fig. 2B were selected incorrectly. Furthermore, the pie charts shown in Fig. 3A were labelled incorrectly; essentially, the pie charts from left to right should have been labelled as ‘Myeloid malignancy’ and ‘Normal’, respectively).

Corrected versions of Figs. 2 and 3 are shown on the next page. The authors would like to thank the Editor of *Oncology Letters* for allowing them the opportunity to publish this corrigendum, and apologize to the readership of the Journal for any inconvenience caused.

## Figures and Tables

**Figure 2.**
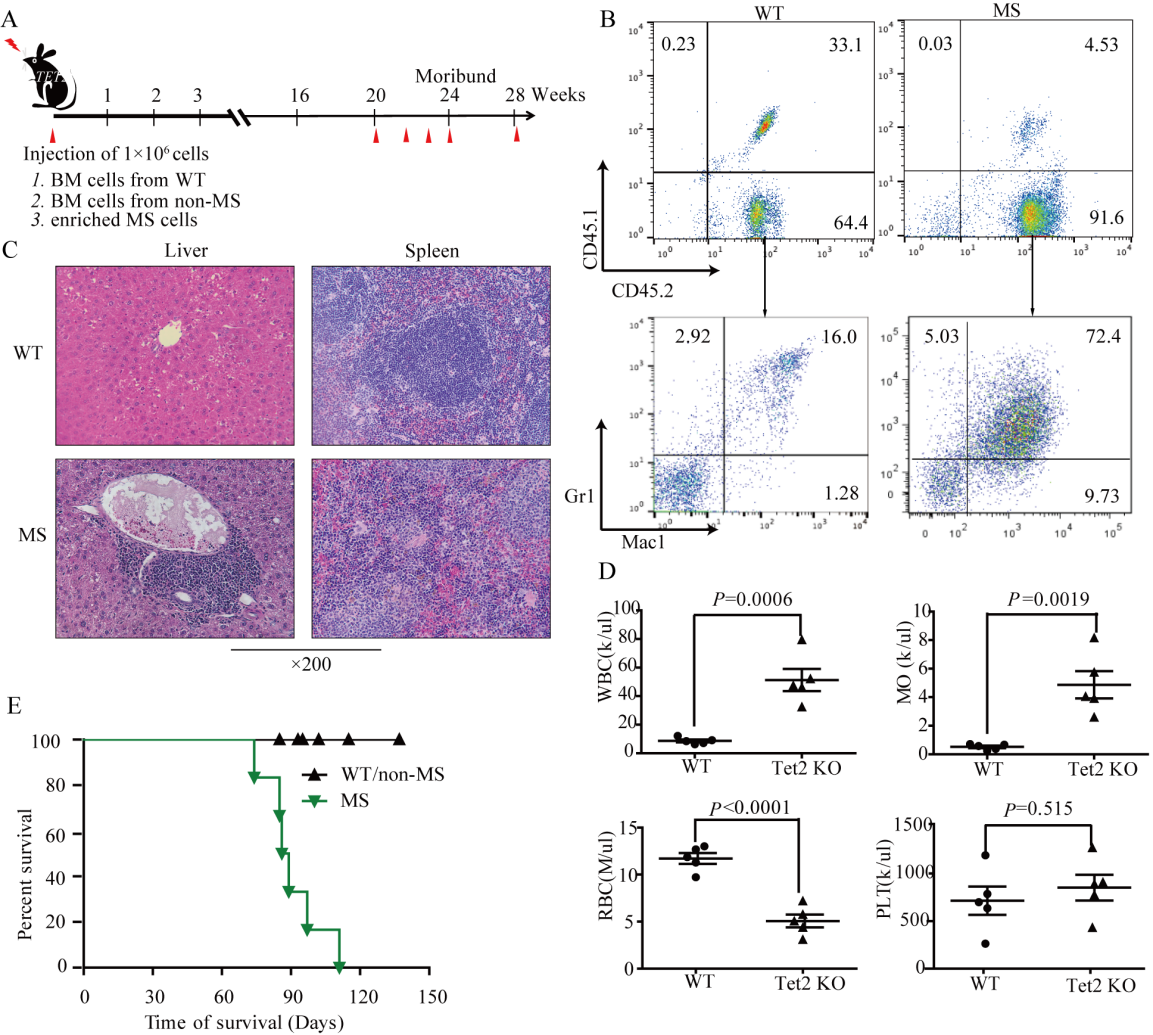
Sarcoma cells in TET2^−/−^ mice are transplantable. (A) Tumor transfer schema. Sarcoma cells (1x10^6^) from a representative TET2^−/−^ mouse with MS or BM cells (1x10^6^) from an age-matched WT mouse were injected into sub-lethally irradiated (800 cGy) recipients (n=5). (B) Flow cytometric analyses of periph- eral blood myeloid lineage (Mac1^+^/Gr1^+^) donor cells (CD45.2^+^) from a mouse receiving BM cells from a WT mouse or sarcoma cells from a TET2^−/−^ mouse with MS mass. (C) Hematoxylin and eosin-stained histological sections of spleen and liver from a representative recipient mouse. Infiltration of a uniform myeloid malignancy cell population was identified in spleen and liver. Infiltrating patterns and cell morphology were similar to those observed in the donor (MS) mouse. These data demonstrate that the recipients receiving MS cells developed a disease similar to their donor mouse. Magnification, x200. (D) Most of the recipients exhibited elevated WBC monocyte, and decreased RBC counts (n=5). (E) Kaplan-Meier survival curve of sub-lethally irradiated recipients transplanted with BM cells (1x10^6^) from a WT mouse or MS cells from TET2^−/−^ mice. WT, wild-type; MS, myeloid sarcoma; BM, bone marrow.

**Figure 3.**
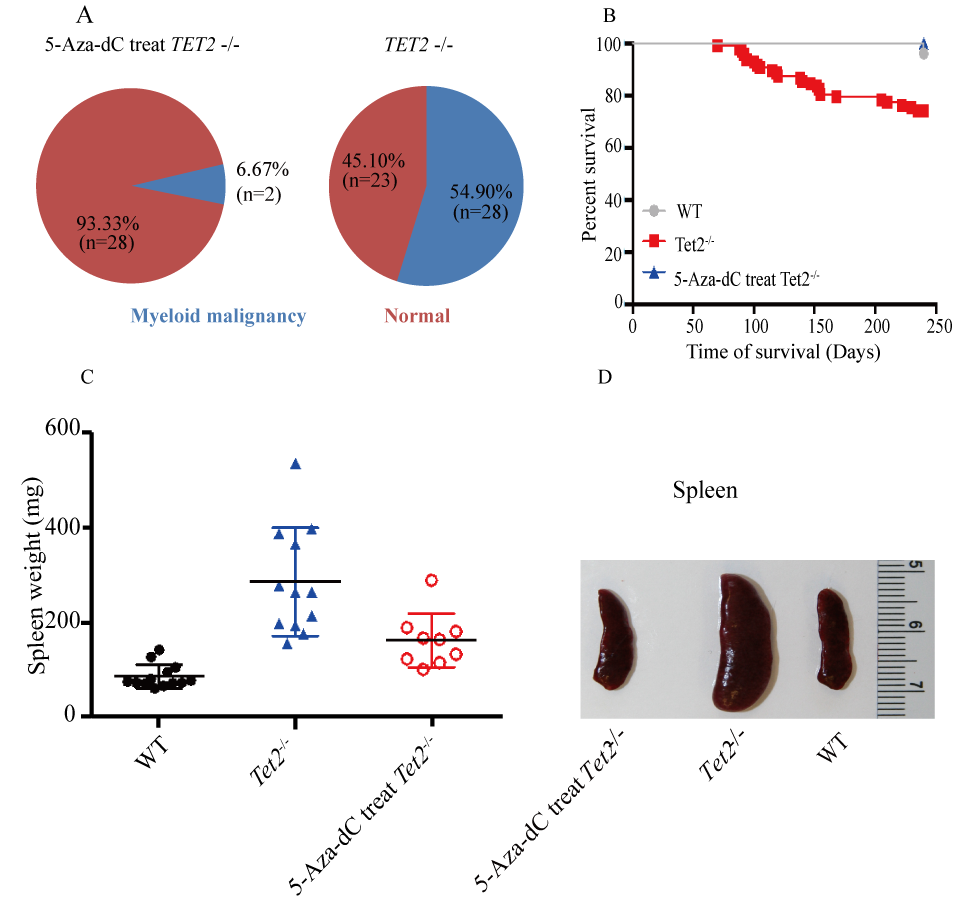
Salvage effect of 5-Aza-dC for TET2^−/−^ myeloid malignancies. (A) Proportions of myeloid malignancies in mice of 5-Aza-dC treated TET2^−/−^ cohorts were lower after half-a-year of (A) follow-up with (B) an improved survival. (C) Weight of spleen from representative TET2^−/−^ mice and TET2^−/−^ mice treated with 5-Aza-dC, as well as age-matched WT mice. (D) Photos of spleen from representative TET2^−/−^ mice and TET2^−/−^ mice with 5-Aza-dC treatment, as well as age-matched WT mice. WT, wild-type.

